# Analysis of 2023 World Health Organization cancer Essential Medicines List and concordance with resource-stratified guidelines

**DOI:** 10.1093/jnci/djaf100

**Published:** 2025-05-23

**Authors:** Brooke E Wilson, Kristin Wright, Manju Sengar, Richard Sullivan, Sallie-Anne Pearson, Michael B Barton, Bishal Gyawali, Elisabeth De Vries, Lorenzo Moja, C S Pramesh, Christopher M Booth

**Affiliations:** Department of Oncology, Queen’s University, Kingston, ON, Canada; Division of Cancer Care and Epidemiology, Queen’s Cancer Research Institute, Kingston, ON, Canada; School of Population Health, Faculty of Medicine and Health, UNSW Sydney, Sidney, NSW, Australia; Department of Oncology, Queen’s University, Kingston, ON, Canada; Department of Medical Oncology, Tata Memorial Centre, Homi Bhabha National Institute, National Cancer Grid, Mumbai, India; Institute of Cancer Policy, King's College London, London, United Kingdom; Department of Oncology, Guy’s and St Thomas’ NHS Foundation Trust, London, United Kingdom; School of Population Health, Faculty of Medicine and Health, UNSW Sydney, Sidney, NSW, Australia; Menzies Centre for Health Policy, University of Sydney, Sidney, NSW, Australia; Faculty of Medicine, University of New South Wales, Sydney, NSW, Australia; Department of Oncology, Queen’s University, Kingston, ON, Canada; Division of Cancer Care and Epidemiology, Queen’s Cancer Research Institute, Kingston, ON, Canada; University Medical Centre Groningen, University of Groningen, Groningen, the Netherlands; Department of Health Product Policy and Standards, World Health Organization, Geneva, Switzerland; Departments of Surgical Oncology and Administration, Tata Memorial Centre, Homi Bhabha National Institute, National Cancer Grid, Mumbai, India; Department of Oncology, Queen’s University, Kingston, ON, Canada; Division of Cancer Care and Epidemiology, Queen’s Cancer Research Institute, Kingston, ON, Canada

## Abstract

**Background:**

The World Health Organization (WHO) Essential Medicines List (EML) and resource-stratified guidelines both prioritize high-value medicines. We compared the 2023 EML cancer medicines list with global cancer statistics, examined the proportion of EML therapies recommended by resource-stratified guidelines, and identified gaps that merit evaluation by the WHO EML Committee.

**Methods:**

We compared the 2023 EML medicines for adult cancers with cancer incidence and mortality data from GLOBOCAN 2022. We cross-referenced the EML with 2 resource-stratified guidelines (National Comprehensive Cancer Network [NCCN] and National Cancer Grid [NCG] of India) and evaluated preferred treatments in resource-stratified guidelines that were not recommended by the EML.

**Results:**

The 2023 EML included 64 cancer medicines for 219 tumor-specific indications. The greatest number of medicines are listed for leukemia (37/219 [17%]) and lymphoma (33/219 [15%]). Although some common cancers (eg, hepatocellular carcinoma) have no EML-listed medicines because of the low clinical benefit, we identified some cancers (eg, esophageal, gastric) with effective therapies that the EML Committee should evaluate for inclusion. Cancers with no listed medicines make up 34% of cancer deaths globally. Among EML indications with an NCCN resource-stratified guideline, 42% (35/84) and 73% (62/84) were recommended by the NCCN Basic and Core Guidelines, respectively. Among EML indications with an NCG resource-stratified guideline, 163 of 196 (83%) and 175 of 196 (89%) were recommended by the NCG Essential and Optimal guidelines, respectively.

**Conclusions:**

We identified some effective medicines that should be evaluated for inclusion in the WHO EML. Prioritization of cancer medicines was similar between the EML and NCG India but discordant between with NCCN resource-stratified guideline.

## Introduction

Prioritizing effective, safe, cost-effective, and affordable health care is a fundamental tenet of universal health coverage and sustainable health systems. Governments must prioritize the access and availability of cancer medicines with the greatest benefit to patients and populations, especially true in resource-limited settings. Although health technology assessments serve as a mechanism to establish cost-effectiveness in high-income countries, they are not available in many low- and middle-income countries (LMIC). A variety of alternative tools have been developed to support decision making.

One of these tools is the World Health Organization (WHO) Essential Medicines List (EML).^1^ Designed for use in both high- and low-income settings, the WHO has produced the EML biennially since 1977, and the most recent iteration was published in 2023.^1^ The EML includes medicines for cancer in addition to other common diseases. Generated by an expert committee following the submission of applications by the oncology community, this list contains medicines considered to be most “essential,” accounting for a range of factors, including clinical effectiveness at the population level, cost, safety, quality of life (QOL), and availability.^1^^,^^2^ In theory, medicines for the most prevalent cancers should be addressed by EML recommendations.

Another tool for prioritization of rational cancer care are resource-stratified guidelines, such as those produced by the National Comprehensive Cancer Network (NCCN)^3^ and the National Cancer Grid (NCG) of India.^4^ Resource-stratified guidelines outline a practical framework for cancer management across settings with different resource constraints by applying available services in sequence based on their benefit and affordability.^3^ Some resource-stratified guidelines, such as those of the NCCN, have been developed for a broad range of tumor types and are designed for global application.^3^^,^^5^ Other country- and region-specific resource-stratified guidelines have been developed, such as those of the NCG. However, no studies have correlated these resource-stratified guidelines with the EML.

Both the EML and resource-stratified guidelines have the potential to influence government health investment policies. However, it is unknown whether the medicines recommended by the EML and resource-stratified guidelines are concordant. Some alignment between the EML and resource-stratified guidelines is desirable; otherwise, the medicines prioritized for government funding based on EML recommendations will be discordant with the medicines that clinicians want to use after consulting treatment guidelines. Prior work has shown convergence in medicines included in the EML and those prioritized by clinicians working in resource-limited settings^6^; however, there was some divergence between the medicines prioritized by clinicians and those included in the NCCN resource-stratified guideline recommendations.^7^ A comprehensive comparison of the EML medicines and treatment recommendations in resource-stratified guidelines is important for examining synergies and exploring areas of discordance and their potential reasons.

The primary objective of this article was to review the 2023 WHO EML for cancer, describe the types of medicines and indications, compare EML-recommended medicines with global cancer incidence and mortality, and identify potential gaps that merit evaluation by the EML Committee. The secondary objective was to examine the proportion of EML cancer medicines concordant with those listed in NCCN and NCG resource-stratified guidelines.

## Methods

We searched the 2023 WHO EML^1^ to identify all medicines related to malignancy, cancer, or oncology. For each listed medicine, we extracted the following information: medicine name, indications, and class of agent (targeted, cytotoxic, supportive, hormone, immunotherapy). Medicines included in the EML are expected to have an overall survival benefit of 4 to 6 months, with improved QOL compared with standard treatments.^8^ Medicines are categorized into core and complementary. Core medicines encompass minimum medicines needed for a health-care system, and complementary medicines encompass those for which specialized health-care workforce, infrastructure, or training are needed. All chemotherapy, targeted small molecules, immunotherapy, and monoclonal antibody medicines are categorized as complementary. We generated a descriptive summary of the characteristics of the EML for cancer compared with GLOBOCAN 2022 population-level data,^9^ which provides cancer incidence and mortality statistics for 36 cancers in 185 countries.

We then cross-referenced and tabulated cancer medicines on the EML with recommendations in the NCCN resource-stratified guideline^3^ and NCG resource-stratified guideline^4^ to determine concordance. Although several resource-stratified guidelines are available, to maintain feasibility we selected 2 representative resource-stratified guidelines: (1) the NCCN, which has the most comprehensive set of resource-stratified guidelines of any international organization, and (2) India’s NCG resource-stratified guidelines to reflect the perspective of an LMIC with a large population, competing health-care needs, wide variation in availability of resources, and expertise in cancer care.^10^ Our analysis focused on the Basic and Core settings for the NCCN resource-stratified guidelines and Essential and Optimal NCG treatment recommendations because we felt that these most accurately aligned with the purpose of the EML. Resource-level definitions are provided in Table S1. The NCCN Basic resources include essential services needed to provide a basic minimum standard of care that improves disease-specific outcomes. The NCCN Core resources include services provided in the Basic setting plus additional services that provide major improvements in disease outcomes (eg, survival) that are not cost prohibitive. The NCG Essential resource-stratified guideline includes recommendations based on the evidence, practicality, the cost of treatment, and the value it offers. The NCG Optimal recommendations are based on evidence as well as cost-effectiveness but may not be widely available because of issues with expertise and infrastructure. Although our analysis focused on adult cancer and the adult EML, the EML does include listings for several pediatric-predominant diseases, including nephroblastoma and retinoblastoma. Recommended medicines for these cancers were extracted from the NCG pediatric resource-stratified guidelines. Finally, we performed a detailed comparison of preferred medicines included in the NCCN Core guidelines and those on the EML to identify any potential cancer medicines that might warrant consideration for inclusion on the EML. All data were extracted by 2 authors (B.E.W. and K.W.), and NCG agreement was verified by a third author (M.S.).

## Results

### Description of the existing EML for cancer

The 2023 WHO EML includes 64 cancer medications for 224 treatment indications. Among the 64 medicines, 36 are cytotoxic, 10 are targeted, 4 are immunomodulators, 9 are hormone or antihormone therapies, and 5 are supportive medicines. Among the 224 indications, 219 are tumor specific and 5 are tumor agnostic (zoledronic acid, allopurinol, rasburicase, filgrastim, and pegfilgrastim).

There are EML-listed medicines for 32 cancer conditions. The greatest number are listed for leukemia (37/219 [17%]) and lymphoma (33/219 [15%]), for which multiple line options are characterized by high response rates and high long-term survival rates at 5 years. Based on GLOBOCAN 2022, leukemia makes up 2.5% of all cancer-related malignancies in adults and 3.2% of all cancer-related deaths, while lymphomas make up 3.3% of all incident cases of cancer in adults and 2.8% of all cancer-related deaths (Table 1). Lung cancer makes up 13% of global cancer incidence and 19% of global cancer deaths. The EML lists 6 medicines for lung cancer. Even with effective therapy, cure rates are relatively low, and lung cancer has one of the highest mortality to incidence ratios (0.73) (Table 1). Breast cancer is the most common malignancy globally, with approximately 2.3 million incident cases annually and a mortality to incidence ratio of 0.29. The EML currently lists 20 medicines for breast cancer, making up 9% of all EML cancer medicines.

**Table 1.** 2021 EML-recommended medicines, stratified by tumor type and compared with GLOBOCAN 2022 incidence, mortality, and mortality to incidence ratios.

Tumor type using GLOBOCAN groupings	EML medicines by tumor type, No. (%) of all EML medicines (219 tumor-specific indications)	Cancer incidence per GLOBOCAN 2022, No.	Cancer mortality per GLOBOCAN 2022, No.	Mortality to incidence ratio
Breast^a^	20 (9)	2 296 840	666 103	0.29
Lung	6 (2.7)	2 480 675	1 817 469	0.73
Colon/rectal^a^	12 (5.5)	1 926 425	904 019	0.47
Prostate	5 (2.5)	1 467 854	397 430	0.27
Stomach	—	968 784	660 175	0.68
Liver	—	866 136	758 725	0.88
Cervical	5 (2.5)	662 301	348 874	0.53
Esophagus	—	511 054	445 391	0.87
Thyroid	—	821 214	47 507	0.06
Bladder	—	614 298	220 596	0.36
Lymphoma		635 858	273 412	0.43
Non-Hodgkin lymphoma	33 (15)	553 389	250 679	
Hodgkin lymphoma	Combined with above	82 469	22 733	
Anaplastic large cell lymphoma	9 (4)			
Pancreas	—	510 992	467 409	0.91
Leukemia	37 (17)	487 294	305 405	0.63
Kidney	—	434 840	155 953	0.36
Corpus uteri	—	420 368	97 723	0.23
Head and neck	5 (2.5)	947 156	482 428	0.51
Lip and oral cavity		389 846	188 438	
Nasopharynx		120 434	73 482	
Oropharynx		106 400	52 305	
Hypopharynx		86 257	40 902	
Salivary glands		55 083	23,942	
Larynx		189 136	103 359	
Melanoma	1 (0.5)	331 722	58 667	0.18
Ovary		324 603	206 956	0.64
Epithelial ovarian	3 (1.5)			
Germ cell tumors/gestational tumors	21 (9.5)			
Central nervous system	3(1.5)	321 731	248 500	0.77
Multiple myeloma	5 (2.5)	187 952	121 388	0.65
Gallbladder	—	122 491	89 055	0.73
Testis (including germ cell tumors)	Encompassed in germ cell tumors under ovary	72 040	9068	0.13
Vulva	—	47 336	18 579	0.39
Penis	—	37 700	13 738	0.36
Kaposi sarcoma	6 (3)	35 813	16 169	0.45
Mesothelioma	—	30 633	25 371	0.83
Vagina	—	18 819	8240	0.44
Other sites/unspecified		1 159 037	810 066	0.70
Retinoblastoma	2 (1)			
Nephroblastoma	4 (2)			
Sarcoma (excluding Kaposi sarcoma)	22 (10)			
Langerhans cell histiocytosis	6 (2.7)			
All cancers, excluding nonmelanoma skin cancer	—	19 165 943	—	—
All sites	—	19 292 789	9 620 190	0.50
Supportive care medications (not tumor specific)	5	N/A	N/A	N/A

Abbreviations: EML = Essential Medicines List; N/A = not applicable.

a In keeping with EML designation, early and metastatic breast, colon, and rectal cancers are considered separately. Thus, if a medicine is recommended in the EML for both early and metastatic breast or colon cancer, this is considered 2 separate indications. In the metastatic setting, rectal cancer is considered with colon cancer as colorectal cancer, although early rectal and colon cancers are considered separately.

**Figure 1.**
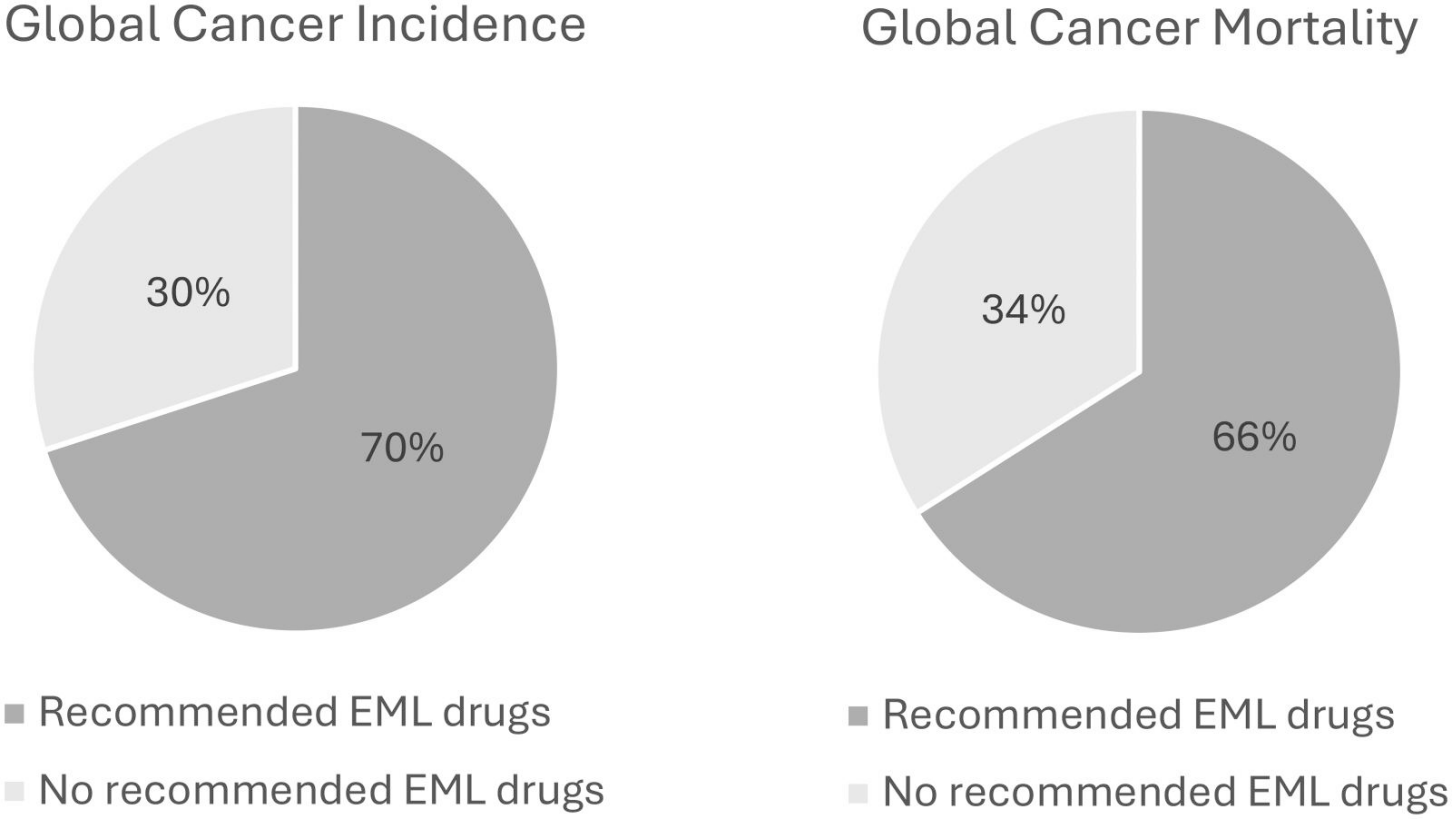
Global cancer incidence and mortality and Essential Medicines List (EML) medicine coverage. In this figure, we exclude the proportion of patients in GLOBOCAN listed as having “other malignancies” due to uncertainty regarding type of cancer and EML drug recommendations. Therefore, the denominator for global cancer incidence is *n* = 18 133 752 and for global cancer mortality is *n* = 8 810 124.

There are several malignancies for which no medicines are recommended on the EML, including stomach, liver, esophagus, thyroid, bladder, pancreas, kidney, uterus, gallbladder, vulva, penis, mesothelioma, and vagina. Together, these cancers make up 30% of incident cancer cases globally (5.4 million/18.1 million, excluding patients with other or unspecified sites). Even with the best available treatment, many of these cancers have high mortality to incidence ratios, and treatments do not meet EML efficacy thresholds (survival prolonged by 4-6 months), justifying their exclusion from the EML. The number of annual deaths attributable to these cancers is 3.0 million, making up 34% of all site-specified cancer-related deaths (Figure 1).

### Agreement of EML and resource-stratified guideline treatment recommendations

The NCCN has generated 64 cancer-specific treatment guidelines, while the NCCN resource-stratified guidelines cover 17 tumor types. The NCG has treatment guidelines for 43 tumor types, all of which are resource stratified. The 2023 EML includes cancer medicines for 32 tumor types (Table S2).

Of the 219 tumor-specific indications on the WHO EML, 135 had no matching NCCN resource-stratified guideline for evaluation (Table 2). Tumor types with cancer medicines included on the EML but not covered by the NCCN resource-stratified guidelines include acute myeloid leukemia, bone cancers, central nervous system tumors, chronic lymphocytic leukemia, chronic myeloid leukemia, gastrointestinal stromal cell tumors, gestational neoplasia, Hodgkin lymphoma, Kaposi sarcoma, cutaneous melanoma, multiple myeloma, ovarian cancer, acute lymphoblastic leukemia, Hodgkin lymphoma, B-cell lymphomas, soft tissue sarcomas, testicular cancer, and nephroblastoma. Among the 84 EML cancer medicine listings with a matching evaluable NCCN resource-stratified guideline, 42% (35/84) were recommended for use by both the EML and the NCCN Basic guidelines, while 74% (62/84) were recommended for use by both the EML and the NCCN Core guidelines. We also identify 89 tumor types for which NCCN resource-stratified guidelines have been developed but there are no EML-supported medicines: bladder, esophageal, gastric, hepatobiliary, kidney, pancreas, thyroid, and uterine.

**Table 2.** EML-supported medicine recommendations and corresponding NCCN and NCG resource-stratified guideline recommendations.^a^

Tumor type	EML-recommended medicine	NCCN Basic	NCCN Core	NCG Essential	NCG Optimal
Breast cancer–early (*n* = 11)	Cyclophosphamide				
	Carboplatin				
	Docetaxel				
	Doxorubicin				
	Fluorouracil				
	Methotrexate				
	Paclitaxel				
	Trastuzumab				
	Anastrozole (alternative: aromatase inhibitor)				
	Leuprorelin (alternative goserelin, triptorelin)^b^				
	Tamoxifen				
Breast cancer–metastatic (*n* = 9)	Capecitabine				
	Cyclophosphamide				
	Docetaxel				
	Doxorubicin				
	Paclitaxel				
	Vinorelbine				
	Trastuzumab				
	Anastrozole (alternative: aromatase inhibitor)				
	Tamoxifen				
Colon cancer–early (*n* = 4)	Fluorouracil				
	Oxaliplatin				
	Calcium folinate				
	Capecitabine				
Rectal cancer–early (*n* = 3)	Fluorouracil				
	Calcium folinate				
	Capecitabine				
Colorectal cancer–metastatic (*n* = 5)	Fluorouracil				
	Oxaliplatin				
	Irinotecan				
	Calcium folinate				
	Capecitabine				
Non–small cell lung cancer (*n* = 7)	Cisplatin				
	Carboplatin				
	Etoposide				
	Gemcitabine				
	Paclitaxel				
	Vinorelbine				
	Erlotinib (alternatives: gefitinib, afatinib)				
Prostate cancer–metastatic (*n* = 5)	Docetaxel				
	Abiraterone (alternative: enzalutamide)				
	Bicalutamide (alternative: flutamide, nilutamide)^c^				
	Leuprorelin (alternatives: goserelin, triptorelin)^c^				
	Prednisolone				
Melanoma (*n* = 1)	Nivolumab (alternative: pembrolizumab)				
Kaposi sarcoma (*n* = 6)	Doxorubicin				
	Bleomycin				
	Paclitaxel				
	Vinblastine				
	Vincristine				
	Pegylated liposomal doxorubicin				
Epithelial ovarian cancer (*n* = 3)	Carboplatin				
	Paclitaxel				
	Gemcitabine				
Cervical cancer (*n* = 3)	Carboplatin				
	Cisplatin				
	Paclitaxel				
Head and neck (*n* = 2)	Carboplatin (as radiation therapy sensitivity)^d^				
	Cisplatin (as radiation therapy sensitivity)^d^				
Nasopharyngeal (*n* = 4)	Carboplatin (as radiation therapy sensitivity)				
	Cisplatin (as radiation therapy sensitivity)				
	Fluorouracil				
	Paclitaxel				
Multiple myeloma (*n* = 8)	Cyclophosphamide				
	Doxorubicin				
	Melphalan				
	Bortezomib				
	Lenalidomide				
	Thalidomide				
	Dexamethasone				
	Prednisolone				
Central nervous system/low-grade glioma (*n* = 5)	Carboplatin				
	Cisplatin				
	Cyclophosphamide				
	Vinblastine				
	Vincristine				
Subependymal giant cell astrocytoma (*n* = 1)	Everolimus				
Nephroblastoma (*n* = 9)	Carboplatin				
	Cyclophosphamide				
	Dactinomycin				
	Doxorubicin				
	Etoposide				
	Ifosfamide				
	Irinotecan				
	Vincristine				
	Mesna				
Retinoblastoma (*n* = 3)	Carboplatin				
	Etoposide				
	Vincristine				
Acute lymphoblastic leukemia (*n* = 16)	Cyclophosphamide				
	Asparaginase				
	Cytarabine				
	Daunorubicin				
	Doxorubicin				
	Etoposide^e^				
	Mercaptopurine				
	Methotrexate				
	Pegaspargase				
	Tioguanine				
	Vincristine				
	Imatinib				
	Dexamethasone				
	Hydrocortisone				
	Methylprednisone				
	Prednisolone				
Acute myeloid leukemia (*n* = 3)	Cytarabine				
	Daunorubicin				
	Etoposide				
Acute promyelocytic leukemia (*n* = 7)	Cytarabine				
	Arsenic trioxide				
	Daunorubicin				
	Mercaptopurine				
	Methotrexate				
	Realgar indigo naturalis formulation				
	All-trans retinoid acid				
Chronic myeloid leukemia (*n* = 4)	Hydroxycarbamide				
	Dasatinib				
	Imatinib				
	Nilotinib				
Chronic lymphocytic leukemia (*n* = 7)	Bendamustine				
	Chlorambucil				
	Cyclophosphamide				
	Fludarabine				
	Ibrutinib				
	Rituximab				
	Prednisolone				
Burkitt lymphoma (*n* = 14)	Calcium folinate				
	Cyclophosphamide				
	Cytarabine				
	Doxorubicin				
	Etoposide				
	Ifosfamide				
	Methotrexate				
	Vincristine				
	Rituximab				
	Dexamethasone				
	Hydrocortisone				
	Methylprednisone				
	Prednisolone				
	Mesna				
Diffuse large B-cell lymphoma (*n* = 5)	Cyclophosphamide				
	Doxorubicin				
	Vincristine				
	Rituximab				
	Prednisolone				
Follicular lymphoma (*n* = 6)	Cyclophosphamide				
	Bendamustine				
	Doxorubicin				
	Vincristine				
	Rituximab				
	Prednisolone				
Anaplastic large cell lymphoma (*n* = 9)	Cyclophosphamide				
	Cytarabine				
	Doxorubicin				
	Etoposide				
	Ifosfamide				
	Methotrexate				
	Vinblastine				
	Prednisone				
	Dexamethasone				
Hodgkin lymphoma (*n* = 9)	Bleomycin				
	Cyclophosphamide				
	Dacarbazine				
	Doxorubicin				
	Etoposide				
	Procarbazine				
	Vinblastine				
	Vincristine				
	Prednisolone				
Osteosarcoma (*n* = 8)	Calcium folinate				
	Carboplatin				
	Cisplatin				
	Doxorubicin				
	Etoposide				
	Ifosfamide				
	Methotrexate				
	Mesna				
Ewing sarcoma (*n* = 7)	Cyclophosphamide				
	Dactinomycin				
	Doxorubicin				
	Etoposide				
	Ifosfamide				
	Vincristine				
	Mesna				
Gastrointestinal stromal tumor (*n* = 1)	Imatinib				
Rhabdomyosarcoma (*n* = 7)	Cyclophosphamide				
	Dactinomycin				
	Ifosfamide				
	Irinotecan				
	Vincristine				
	Vinorelbine				
	Mesna				
Ovarian granulosa cell tumor (*n* = 8)	Bleomycin				
	Carboplatin				
	Cisplatin				
	Etoposide				
	Ifosfamide				
	Paclitaxel				
	Vinblastine				
	Mesna				
Testicular germ cell tumor (*n* = 7)	Bleomycin				
	Carboplatin				
	Cisplatin				
	Etoposide				
	Ifosfamide				
	Vinblastine				
	Mesna				
Gestational trophoblastic neoplasia (*n* = 6)	Calcium folinate				
	Cyclophosphamide				
	Dactinomycin				
	Etoposide				
	Methotrexate				
	Vincristine				
Langerhans cell histiocytosis (*n* = 6)	Prednisone				
	Cytarabine				
	Mercaptopurine				
	Methotrexate				
	Vinblastine				
	Vincristine				
Tumor lysis syndrome (*n* = 2)	Allopurinol	—	—	—	—
	Rasburicase	—	—	—	—
Bone metastases (*n* = 1)	Zoledronic acid	—	—	—	—
Bone marrow support (*n* = 2)	Filgrastim	—	—	—	—
	Pegfilgrastim	—	—	—	—

Abbreviations: EML = Essential Medicines List; NCCN = National Comprehensive Cancer Network; NCG = National Cancer Grid.

a White indicates that a medicine is recommended by the guideline, light gray indicates that a medicine is not recommended by the guideline, dark gray indicates that there was no evaluable guideline.

b Leuprolide is supported in NCCN Core and NCGI Optimal guidelines through the inclusion of the statement that ovarian suppression or ablation is supported, although the specific medicines are not provided.

c Bicalutamide and leuprolide are supported in NCG Essential and Optimal guidelines through the inclusion of the statement that androgen-deprivation therapy is supported, although the specific medicines are not provided.

d Carboplatin and cisplatin are listed in the NCCN guidelines for metastatic disease without radiation therapy, but the EML clearly restricts the indication in this context to be carboplatin and cisplatin in the curative setting in combination with radiation.

e Etoposide is listed in the NCCN Core setting for relapsed or refractory Philadelphia chromosome–negative acute lymphoblastic leukemia.

Of the 219 tumor-specific medicines on the 2023 EML, 23 had no evaluable resource-stratified Essential guideline for review. Tumor types with an EML listing and no matching evaluable NCG resource-stratified guideline included Kaposi sarcoma, cutaneous melanoma, subependymal giant cell astrocytoma, and gestational trophoblastic neoplasia. There was a high level of concordance between the EML and matching NCG resource-stratified guidelines, with 163 of 196 (83%) medicines recommended for use by both the EML and the NCG Essential guideline and 175 of 196 (89%) medicines recommended for use by both the EML and the NCG Optimal listing.

### Medicines that merit further evaluation by the EML Committee

We identified several medicines listed in the NCCN Core guidelines that might warrant consideration by the EML Committee (Table S3). These medicines include some commonly available low-cost hormone therapies (eg, tamoxifen for uterine cancer) and older but widely available and effective chemotherapeutic agents (eg, oxaliplatin and 5-fluorouracil [5-FU] for gastroesophageal cancers; carboplatin and paclitaxel for uterine cancer). In total, we identified 8 tumors for which additional medicines could be considered for listing on the EML (Table S3). If the EML Committee were to include the additional suggested medicines within their recommendations, the proportion of patients who could be treated would rise from approximately 70% to 84% of patients with a cancer diagnosis. The number of deaths attributable to cancers with no recommended medicines on the EML would fall from 3.0 million to 1.6 million. Although each potential medicine would require a comprehensive evaluation by the EML Committee, we provide 2 case examples in the palliative setting for illustrative purposes. We briefly evaluate them across the 5 principal EML domains (efficacy, toxicity, QOL, availability, and cost-effectiveness) (Table 3).

**Table 3.** Case study examples of older medicines not included on the EML.

Variable	Case 1	Case 2
Tumor type	Gastroesophageal cancer	Bladder cancer
Medicines	5-Fluorouracil and oxaliplatin	Cisplatin and gemcitabine
Treatment intent	Palliative	Palliative
Line of therapy	First	First
Approximate median survival—no treatment	3-6 mo	6 mo
Approximate median survival—with treatment	7-12 mo	14 mo
**Brief evaluation of EML criteria**		
Efficacy	>4-9 mo survival gain	>8 mo survival gain
Availability	Already included for colon and rectal cancer	Already included for non–small cell lung cancer, head and neck cancers, breast cancer
QOL	This combination has been shown to maintain QOL in patients with advanced or metastatic gastroesophageal cancer^23^	This combination has been shown to maintain QOL in patients with advanced or metastatic bladder cancer, with improvements demonstrated in emotional functioning and pain^13^
Toxicity	Acceptable toxicity profiles in other tumor types; no evidence of differential tolerance among patients with gastroesophageal cancer	Acceptable toxicity profiles in other tumor types; no evidence of differential tolerance among patients with bladder cancer
Cost-effectiveness	Formal studies required; however, these are relatively inexpensive medicines with clinically meaningful gains and are likely to be cost-effective in most settings	Formal studies required; however, these are relatively inexpensive medicines with clinically meaningful gains and are likely to be cost-effective in most settings

Abbreviations: EML = Essential Medicines List; QOL = quality of life.

In case 1, we note that 5-FU and oxaliplatin are listed as essential medicines for metastatic colorectal cancer, but they are not currently listed for esophageal and gastric cancer. Median overall survival for metastatic gastroesophageal cancer with the best supportive care is 3 to 6 months compared to 7 to 12 months for patients receiving 5-FU and oxaliplatin,^11^^,^^12^ for an estimated net survival gain of 4 to 6 months. The safety and tolerability of these medications are acceptable. Quality of life criteria are likely to be met given the positive EML evaluation of the same medicines for other tumor types. The availability is demonstrated because oxaliplatin and 5-FU are off patent and accessible in most LMIC. These medicines are unlikely to represent a major financial burden for LMIC, but dedicated cost-effectiveness analyses are required.

In case 2, we note that gemcitabine and cisplatin are standard of care for metastatic bladder cancer in most treatment guidelines (including NCCN Core) and are supported by the EML for several other tumor types. However, they are not listed for bladder cancer in the current EML. Median overall survival without treatment is approximately 6 months compared with 14 months with gemcitabine and cisplatin,^13^ an estimated median survival gain that surpasses the 6-month EML threshold. Gemcitabine and cisplatin also have a well-known safety and tolerability profile and are widely available in LMIC. Although cost-effectiveness studies focused on bladder cancer in LMIC are limited, these medicines are available as generics and are unlikely to pose financial challenges.

## Discussion

The 2023 adult EML encompasses 32 cancers, including some of the most common cancers globally. However, 34% of cancer deaths occur in tumor types that do not have any EML-listed cancer medicines, including some highly prevalent tumors (eg, hepatocellular carcinoma). For many of these cancers, approved medicines have marginal clinical gains, making their exclusion from the EML justifiable. However, we also identified effective and accessible medications that may warrant consideration so that their indications are extended to cancer types not included in the 2023 EML (Table S3). In fact, use of these medicines in select cancers appears to meet WHO criteria for inclusion on the EML. Although we found discordance between cancer medicines on the EML and those recommended in NCCN resource-stratified guidelines, there was good concordance between cancer medicines on the EML and NCG resource-stratified guidelines.

We identified several effective and accessible chemotherapeutic agents and hormone therapies for common cancers in LMIC that may warrant consideration by the EML Committee. The EML Committee considers proposals put forward by members of the cancer community, independent organizations, and industry for evaluation. Most recent EML submissions have been for new, often expensive, systemic anticancer medicines,^14^ and many older, off-patent chemotherapeutics may not have been submitted for review, presumably due to the costs and time required to prepare submissions. This situation highlights an important challenge in the EML submission and evaluation process, whereby only treatments put forth by invested parties can be considered; therefore, treatments with relevant commercial interests could be privileged over older, off-patent (and effective) medicines. Potential solutions to this challenge include a call to the cancer community for submission of older medicines for evaluation or a reevaluation of the mechanisms that trigger an EML medicine review.

In contrast to the absence of some common cancers from the EML list, we found that other cancer types that make up a relatively small proportion of the global burden of cancer are heavily represented, such as leukemia and lymphoma. These cancers require multiple medicines (generally ranging from 4 to 6) for any given treatment protocol and have high cure rates with appropriate therapy, allowing them to meet key criteria for inclusion on the EML.^2^

When the EML was developed in 1977, medicines were listed without target indications. Over time, the EML has specified the disease for which each medicine is considered appropriate. For example, cytarabine was first added to the EML in 1979 without a clear tumor indication, followed by specifications for lymphoma and acute lymphoblastic leukemia in 2011 and acute myeloid leukemia in 2015. Therefore, the current EML listings can be considered prescriptive regarding the tumors on which medicines should be used.^15^ However, the EML does not provide any guidance on how and when these medications should be used. Furthermore, the EML lists medications only as single agents rather than as combination regimens. Consequently, clinicians often look to guidelines (including resource-stratified guidelines) for information about treatment options.^16^^,^^17^ Some alignment between the EML and resource-stratified guidelines is desirable; otherwise, medicines prioritized by governments and policymakers based on the EML may not align with medicines that clinicians are looking to use after consulting treatment guidelines. Although we believe that it is beyond the scope of the EML Committee to recommend regimens, we encourage future iterations of the EML to consider whether and how the recommended drugs are typically combined in treatment regimens.

Potential explanations for the differences in recommendations between resource-stratified guidelines and the EML include conflicts of interest among guideline participants and the tumor-focused approach of guidelines compared with the magnitude of the benefit-focused approach the EML employs. The composition of the teams making recommendations also differs. The EML Committee includes clinicians from diverse backgrounds representing all 6 WHO regions. The NCG guidelines are developed by clinicians, mostly oncologists, working in a resource-constrained setting, while the NCCN guidelines are generated predominantly by clinicians from high-income countries.^18^ We also recognize that resource-stratified guidelines have not been supported by the WHO, although the WHO proposed in 2002 that national cancer control activities be based on the resource realities of a given country, categorized as low, medium, or high.^19^

The EML and resource-stratified guidelines are only 1 component of several policy levers required to develop and manage an effective and affordable national anticancer therapy program. Tools predicting the number of patients who meet criteria for treatment; the required medicine quantities; costs; workforce requirements; nursing and pharmacy needs; and the magnitude of clinical benefit relative to other interventions, such as surgery and radiation therapy, are needed. Recent work merging resource-stratified guidelines with population-level data regarding cancer incidence from GLOBOCAN has led to interactive models to estimate treatment demands, costs, and medicine procurement needs for a wide range of countries and resource availability for both breast and colon cancer^20^^,^^21^ as well as for pediatric tumors.^22^ Work to develop a similar tool for the EML is ongoing.

Our study has limitations. To increase the availability of medicines for childhood malignancies, the EML includes several rare tumor types (eg, nephroblastoma) not typically seen in adults and not specifically addressed in the GLOBOCAN 2022 data. These malignancies would have been included in the “other” category. In estimating the proportion of patients with cancer for whom there is an EML-recommended medicine, we excluded the proportion of patients with “other” or unspecified cancers. Therefore, the crude number of patients with cancer with an EML-supported medicine may be slightly higher than our estimate. We also acknowledge that for clinical scenarios in which recommendations were directly compared between resource-stratified guidelines and the EML, differences in recommendations might be justifiable if the evaluation criteria differ substantially. Finally, we did not consider other high-quality resource-stratified guidelines (eg, American Society of Clinical Oncology). Our study also has several key strengths. This is the first systematic evaluation of the WHO EML being correlated with globally applicable resource-stratified guidelines and with the global burden of cancer incidence. We were able to demonstrate the concordance of the NCG resource-stratified guidelines with the EML, emphasizing the importance of practical guidelines being developed locally and regionally by clinicians working in resource-constrained settings.

The inclusion of essential medicines targeting rarer cancers on the EML could be perceived as unbalanced compared with the smaller numbers of essential medicines for common cancers. This imbalance is due in part to the substantial clinical benefits of these therapies, relatively low total budget impact due to low incidence, and the associated incentives promoted by orphan drug legislation. However, this landscape analysis also identifies some effective medicines for common cancers that are not currently listed and should be formally evaluated by the EML Committee. This analysis may be useful to organizations such as NCCN and the NCG as they develop future iterations of resource-stratified guidelines and consider how their recommendations intersect with medicines recommended by the WHO EML Committee. The divergent recommendations we observed, particularly among the NCCN resource-stratified guidelines, highlight the challenges faced by governments, policymakers, and clinicians as they select medications for prioritization in the EML and for clinical use. Building synergies across these tools is an important step in reducing conflicting information being introduced into the health system, potentially leading to confusion and wasted resources.

## Supplementary Material

djaf100_Supplementary_Data

## Data Availability

The data used in this work have been drawn from multiple publicly available sources and are referenced accordingly.
