# Supplementary material for: Analysis of 2023 World Health Organization cancer Essential Medicines List and concordance with resource-stratified guidelines
Source: J Natl Cancer Inst. 2025 May 23;117(10):2010–20. doi: 10.1093/jnci/djaf100 (PMC12505124; doi:10.1093/jnci/djaf100)
Supplement: djaf100_Supplementary_Data [file djaf100_supplementary_data.zip › EML supplementals PDF.pdf]

Supplementary Materials for : “Analysis of 2023 WHO Cancer Essential Medicines List and Concordance with Resource Stratified Guidelines”

1. Supplementary Table 1: Resources stratified guideline definitions- NCCN and NCG India

2. Supplementary Table 2: Availability of NCCN guidelines, NCCN and NCGI RSG and EML medicines by tumour type (green= available/indication, red = not available/no indication)

3. Supplementary Table 3: Detailed evaluation of medicines included in NCCN guidelines but excluded for the EML to identify first line options that may warrant further evaluation by the EML Committee

Supplementary Table 1: Resources stratified guideline definitions- NCCN and NCG India

**NCCN**	
**Basic**	Basic Resources include essential services needed to provide basic minimal standard of care that improves disease-specific outcomes
**Core**	Core Resources include services provided in the Basic Resources Framework plus additional services that provide major improvements in disease outcomes (eg, survival) and that are not cost prohibitive.
**Enhanced**	Enhanced Resources include services provided in the Core Resources Framework plus additional services that provide lesser improvements in disease outcomes and/or services that provide major improvements in disease outcomes but are cost prohibitive in lower-resource settings.
**Maximal**	The NCCN Guidelines are evidence-based, consensus-driven recommendations made by the NCCN Guidelines panels. They include services provided in the Enhanced Resources Framework plus additional services that provide minor improvements in disease outcomes, interventions that are cost prohibitive in lower-resource settings, and/or services that do not provide improvement in disease outcomes but are desirable services.
**NCG India**	
**Essential**	Defined as recommendations based on the evidence, practicality (wide availability of expertise and infrastructure) as well as the cost or treatment and the value it offers. If centres do not have the capabilities to implement these, they should refer patients to a higher centre
**Optimal**	Defined as recommendations based on both evidence as well as cost effectiveness, but may not be widely available because of issues with expertise and infrastructure
**Optional**	Defined as one which would reflect the state of the art, and base its recommendations purely on the available evidence with no consideration for cost effectiveness

Supplementary Table 2: Availability of NCCN guidelines, NCCN and NCGI RSG and EML medicines by tumour type (green= available/indication, red = not available/no indication)

	NCCN guidelines (n=64)	NCCN RSG (n=17)	NCGI guidelines (n=43)	NCGI RSG (n=43)	EML indication (N=28)
Acute Lymphoblastic Leukaemia					
Acute Myeloid Leukaemia					
Ampullary Adenocarcinoma					
Anal Carcinoma					
Basal Cell Skin Cancer					
B cell lymphomas					
Biliary Tract Cancers					
Bladder Cancer					
Bone Cancer					
Breast Cancer					
CNS Cancers					
Cervical Cancer					
CLL/SLL					
Chronic Myeloid Leukaemia					
Colon Cancer					
Dermatofibrosarcoma protuberans					
Esophageal/Esophagogastric Junction Cancers					
Gastric					
Gastrointestinal Stromal Tumors					
Gestational Trophoblastic neoplasia					
Hairy cell leukemia					
Head and Neck Cancers					
Hepatobiliary Cancers					
Hepatocellular Carcinoma					
Histiocytic Neoplasma					
Hodgkin Lymphoma					
Kaposi Sarcoma					
Kidney Cancer					
Melanoma – Cutaneous					
Melanoma- Uveal					

Merkel Cell Carcinoma					
Mesothelioma- Peritoneal					
Mesothelioma- Pleural					
Multiple Myeloma					
Myelodysplastic Syndromes					
Myeloid/Lymphoid neoplasms with eosinophilia and TK gene fusions					
Myeloproliferative neoplasms					
Neuroendocrine and adrenal tumors					
Non-Small Cell lung cancer					
Occult Primary					
Ovarian Cancer/Fallopian Tube/Primary Peritoneal					
Pancreatic adenocarcinoma					
Pediatric ALL					
Pediatric Aggressive mature B cell lymphoma					
Pediatric CNS					
Pediatric Hodgkin Lymphoma					
Penile Cancer					
Primary Cutaneous Lymphoma					
Prostate Cancer					
Rectal Cancer					
Small Bowel Adenocarcinoma					
Small Cell Lung cancer					
Soft Tissue Sarcoma					
Squamous cell skin cancer					
Systemic light chain amyloidosis					
Systemic mastocytosis					
T cell lymphomas					
Testicular Cancer					
Thymomas and Thymic Carcinomas					
Thyroid Carcinoma					

Uterine Neoplasms					
Vulvar Cancer					
Waldenstrom Macroglobulinemia/ Lymphoplastmocytic lymphoma					
Wilms Tumor (nephroblastoma)					

Supplementary Table 3: Detailed evaluation of medicines included in NCCN guidelines but excluded for the EML to identify first line options that may warrant further evaluation by the EML Committee

Medicines highlighted in bold are those where there is disagreement between the EML and the NCCN Core Guideline.

Tumor Type	EML recommended medicine	NCCN recommended medicines (Category 1 – Core)	Summary Comments	Suggestion to EML
**Breast cancer – early (n=11)**	Cyclophosphamide<br>Carboplatin<br>Docetaxel<br>Doxorubicin<br>Fluorouracil<br>Methotrexate<br>Paclitaxel<br>**Trastuzumab**<br>Anastrozole<br>Leuprorelin<br>Tamoxifen	Doctaxel/cyclophosphamide<br>Doxorubicin/cyclophosphamide +/- docetaxel/paclitaxel<br>Cyclophosphamide/methotrexate/fluorouracil<br>Paclitaxel/carboplatin<br>**Epirubicin**/cyclophosphamide<br>Anastrozole<br>Leuprorelin<br>Tamoxifen	All preferred regimens in NCCN guideline are available through EML except trastuzumab for HER2 positive breast cancer.<br><br>Epirubicin is not included in EML, though doxorubicin is a valid substitute.	No change
**Breast Cancer-Metastatic (n=9)**	Capecitabine<br>Cyclophosphamide<br>Docetaxel<br>Doxorubicin<br>Paclitaxel<br>Vinorelbine<br>**Trastuzumab**<br>Anastrozole<br>Tamoxifen	Capecitabine<br>Cyclophosphamide<br>Docetaxel<br>Doxorubicin<br>Paclitaxel<br>Vinorelbine<br>Anastrozole<br>**Exemestane**<br>**Letrozole**<br>**Fulvestrant**<br>Tamoxifen	Additional endocrine therapy options in the Core NCCN missing from the EML include letrozole and exemestane, but anastrozole is equivalent and is listed.<br><br>Other missing medicines from EML is fulvestrant.<br><br>In contrast, EML includes trastuzumab which is not listed in NCCN	No change
**Colon cancer – Early (n=4)**	Fluorouracil<br>Oxaliplatin<br>Calcium folinate<br>Capecitabine	5-FU +calcium folinate<br>5-FU/oxaliplatin/calcium folinate<br>Capecitabine<br>Capecitabine/oxaliplatin	Medicine availability for first-line options is matched between NCCN and EML	No change
**Rectal cancer-Early (n=3)**	Fluorouracil<br>Calcium folinate<br>Capecitabine	5-FU +calcium folinate<br>Capecitabine	Medicine availability for first-line options is matched between NCCN and EML	Consider the evaluation of oxaliplatin
**Colorectal cancer-Metastatic (n=5)**	Fluorouracil<br>Oxaliplatin<br>Irinotecan<br>Calcium folinate<br>Capecitabine	5-FU +calcium folinate<br>5-FU/oxaliplatin/calcium folinate<br>Capecitabine<br>Capecitabine/oxaliplatin<br>5FU/Irinotecan/calcium folinate	Medicine availability for first line options is matched between NCCN and EML	No change
**NSCLC (n=6)**	**Cisplatin**<br>**Etoposide**<br>**Gemcitabine**<br>**Paclitaxel**<br>**Vinorelbine**<br>**Erlotinib (or other TKI)**	No guideline supported systemic therapy in Core setting	-	No change
	Docetaxel	Docetaxel/ADT		

**Prostate Cancer-metastatic (n=5)**	Abiraterone/Enza<br>Bicalutamide (alt nilutamide, flutamide) (ADT)<br>Leuprolide (alt triptorelin, goserelin) (ADT)<br>Prednisolone (CS)	Abiraterone/ADT<br>Docetaxel/Abiraterone/ADT<br>ADT options: Nilutamide, Flutamide, **Ketoconazole/hydrocortisone,** Goserelin, Triptorelin, Leuprorelin<br>Steroid options: **Dexamethasone, hydrocortisone**, prednisone	Although the NCCN guidelines include a more extensive list of ADT options, the preferred treatments in NCCN guidelines can be provided using medicines listed in the EML	Consider evaluation of alternative ADT agents and/or steroids to EML inclusion list
**Melanoma (n=1)**	**Nivolumab (alt pembrolizumab)**	No available guideline	-	No change
**Kaposi Sarcoma (n=6)**	**Doxorubicin**<br>**Bleomycin**<br>**Paclitaxel**<br>**Vinblastine**<br>**Vincristine**<br>**Pegylated liposomal doxorubicin**	No available guideline	-	No change
**Epithelial Ovarian Cancer (n=3)**	**Carboplatin**<br>**Paclitaxel**<br>**Gemcitabine**	No available guideline	-	No change
**Cervical Cancer (n=3)**	Carboplatin<br>Cisplatin<br>Paclitaxel	Carboplatin<br>Cisplatin<br>Carboplatin+paclitaxel<br>Cisplatin + paclitaxel<br>Carboplatin + **etoposide**<br>Cisplatin + **etoposide**	Etoposide is listed in NCCN core guidelines for small-cell cervical cancer. Etoposide is not included in EML for this indication, but is included in EML for other indications. Given that other agents for management of small-cell cervical cancer, such as carboplatin and cisplatin, are included, this may warrant reconsideration by the EML.	Consider evaluation of etoposide
**Head and neck (n=2)**	Carboplatin (as RT sens)<br>Cisplatin (as RT sens)	Carboplatin<br>Cisplatin<br>**Paclitaxel**<br>**Docetaxel**<br>**Fluorouracil**<br>**Methotrexate**<br>**Capecitabine**	In the current EML, palliative chemotherapy options such as carboplatin and cisplatin are not included. These are specified as only for treatment in combination with radiation. However, these medicines are available and already included in guidelines for other indications and are low cost. These medicines may warrant reconsideration by the EML	Consider evaluation of carboplatin and cisplatin for metastatic disease (for use without radiation).
**Nasopharyngeal (n=4)**	**Carboplatin (as RT sens)**<br>**Cisplatin (as RT sens)**<br>**Fluorouracil**<br>**Paclitaxel**	No systemic therapy recommended in core resources	No systemic therapy recommended in core resources	No change

**Multiple myeloma (n=8)**	**Cyclophosphamide**<br>**Doxorubicin**<br>**Melphalan**<br>**Bortezomib**<br>**Lenalidomide**<br>**Thalidomide**<br>**Dexamethasone**<br>**Prednisolone**	No available guideline	-	No change
**CNS tumors (n=5)**	**Carboplatin**<br>**Cisplatin**<br>**Cyclophosphamide**<br>**Vinblastine**<br>**Vincristine**	No available guideline	-	No change
**Subependymal giant cell astrocytoma (n=1)**	**Everolimus**	No available guideline	-	No change
**Nephroblastoma (n=9)**	**Carboplatin**<br>**Cyclophosphamide**<br>**Dactinomycin**<br>**Doxorubicin**<br>**Etoposide**<br>**Ifosfamide**<br>**Irinotecan**<br>**Vincristine**<br>**Mesna**	No available guideline	-	No change
**Retinoblastoma (n=3)**	**Carboplatin**<br>**Etoposide**<br>**Vincristine**	No available guideline	-	No change
**Acute lymphoblastic leukaemia (n=16)**	Cyclophosphamide<br>Asparaginase<br>Cytarabine<br>Daunorubicin<br>**Doxorubicin**<br>**Etoposide**<br>Mercaptopurine<br>Methotrexate<br>**Pegasparaginase**<br>**Tioguanine**<br>Vincristine<br>Imatinib (TKI)<br>Dexamethasone (CS)<br>Hydrocortisone (CS)<br>Methylprednisolone (CS)<br>Prednisolone (CS)	(TKI) + Cyclophosphamide + Vincristine + Prednisone + Daunorubicin<br><br>Asparaginase<br><br>Cytarabine + Methotrexate + Corticosteroid<br><br>Methotrexate + Mercaptopurine + Vincristine/Prednisone<br><br>Methotrexate<br><br>Vincristine<br><br>TKI + Vincristine + dexamethasone<br><br>Vincristine + Dexamethasone<br><br>Imatinib (TKI) + CS<br>**Dasatinib (TKI) + CS**<br>**Nilotinib (TKI) + CS**<br>Dexamethasone (CS)<br>Hydrocortisone (CS)<br>Methylprednisolone (CS)	EML includes some additional chemotherapeutic agents, while NCCN guidelines include some additional targeted agents. However, first-line treatments are feasible with the provided listed EML medicines	No change

		Prednisolone (CS)		
**AML (n=3)**	**Cytarabine**<br>**Daunorubicin**<br>**Etoposide**	No available guideline	-	No change
**APML (n=7)**	**Cytarabine**<br>**Arsenic trioxide**<br>**Daunorubicin**<br>**Mercaptopurine**<br>**Methotrexate**<br>**Realgar indigo naturalis formulation**<br>**ATRA**			
**CML (n=4)**	**Hydroxycarbamide**<br>**Dasatinib**<br>**Imatinib**<br>**Nilotinib**	No available guideline	-	No change
**CLL (n=7)**	**Bendamustine**<br>**Chlorambucil**<br>**Cyclophosphamide**<br>**Fludarabine**<br>**Ibrutinib**<br>**Rituximab**<br>**Prednisolone**	No available guideline	-	No change
**Burkitt's Lymphoma (n=14)**	**Calcium folinate**<br>Cyclophosphamide<br>Cytarabine<br>Doxorubicin<br>Etoposide<br>Ifosfamide<br>Methotrexate<br>Vincristine<br>Dexamethasone<br>Rituximab<br>**Hydrocortisone**<br>**Methylprednisone**<br>**Prednisolone**<br>**Mesna**	Cyclophosphamide + intrathecal Methotrexate + high-dose Methotrexate +Vincristine + Cytarabine<br><br>Cyclophosphamide + Doxorubicin + Vincristine + intrathecal Methotrexate + high-dose Methotrexate + Rituximab<br><br>Rituximab + Ifosfamide + Cytarabine + Etoposide + intrathecal Methotrexate<br><br>Etoposide + Prednisone + Vincristine + Cyclophosphamide + Doxorubicin + Rituximab<br><br>Rituximab + Ifosfamide + **Carboplatin** + Etoposide + intrathecal Methotrexate<br><br>Rituximab + **Gemcitabine** + Dexamethasone + **Cisplatin**<br><br>High-dose Cytarabine + Rituximab	Preferred first line options is available with current listed EML medicines.	No change
**DLBCL (n=5)**	Cyclophosphamide<br>Doxorubicin<br>Vincristine<br>Rituximab<br>Prednisolone	Rituximab + Cyclophosphamide + liposomal Doxorubicin + Vincristine + Prednisone	Sufficient medicines listed on EML to provide first-line treatment.	No change

		Rituximab + Cyclophosphamide + Doxorubicin + Vindesine + **Bleomycin** + Prednisone<br><br>Rituximab + Cyclophosphamide + Etoposide + Vincristine + Prednisone<br>Rituximab + Cyclophosphamide + liposomal Doxorubicin + Vincristine + Prednisone<br><br>Etoposide + Prednisone + Vincristine + Cyclophosphamide + Doxorubicin + Rituximab		
**Follicular lymphoma (n=6)**	**Cyclophosphamide**<br>**Bendamustine**<br>**Doxorubicin**<br>**Vincristine**<br>**Rituximab**<br>**prednisolone**	No available guideline	-	No change
**Hodgkin's lymphoma (n=9)**	**Bleomycin**<br>**Cyclophosphamide**<br>**dacarbazine**<br>**Doxorubicin**<br>**Etoposide**<br>**Procarbazine**<br>**Vinblastine**<br>**Vincristine**<br>**Prednisolone**	No available guideline	-	No change
**Osteosarcoma (n=8)**	**Calcium folinate**<br>**Carboplatin**<br>**Cisplatin**<br>**Doxorubicin**<br>**Etoposide**<br>**Ifosfamide**<br>**Methotrexate**<br>**Mesna**	No available guideline	-	No change
**Ewing's sarcoma (n=6)**	**Cyclophosphamide**<br>**Dactinomycin**<br>**Doxorubicin**<br>**Etoposide**<br>**Ifosfamide**<br>**Mesna**	No available guideline	-	No change
**GIST (n=1)**	**Imatinib**	No available guideline	-	No change
**Rhabdomyosarcoma (n=7)**	**Cyclophosphamide**<br>**Dactinomycin**<br>**Ifosfamide**<br>**Irinotecan**<br>**Vincristine**<br>**Vinorelbine**<br>**Mesna**	No available guideline	-	No change
**Ovarian GCT (n=8)**	**Bleomycin**<br>**Carboplatin**<br>**Cisplatin**<br>**Etoposide**<br>**Ifosfamide**	No available guideline	-	No change

	**Paclitaxel**<br>**Vinblastine**<br>**Mesna**			
**Testicular GCT (n=9)**	**Bleomycin**<br>**Carboplatin**<br>**Cisplatin**<br>**etoposide**<br>**Ifosfamide**<br>**Vinblastine**<br>**Mesna**	No available guideline	-	No change
**Gestational trophoblastic neoplasia (n=6)**	**Calcium folinate**<br>**Cyclophosphamide**<br>**Dactinomycin**<br>**Etoposide**<br>**Methotrexate**<br>**Vincristine**	No available guideline	-	No change
**Bladder Cancer**	No EML-listed medicines for this indication	**Cisplatin+gemcitabine**<br>**Carboplatin/gemcitabine**<br>**Paclitaxel/gemcitabine**<br>**gemcitabine**	Unable to treat metastatic bladder cancer at present based on existing EML listing. Cisplatin, gemcitabine, carboplatin, and paclitaxel are commonly used cheap older chemotherapeutics listed elsewhere in the EML, but not included in the bladder cancer indication.	Consider listing for cisplatin, gemcitabine, carboplatin and paclitaxel for bladder cancer
**Biliary Tract Cancers**	No EML-listed medicines for this indication	**Capecitabine**<br>**5-Fluorouracil/oxaliplatin/calcium folinate**<br>**Capecitabine/oxaliplatin**<br>**Gemcitabine/capecitabine**<br>**5-Fluorouracil/calcium folinate**<br>**Gemcitabine/oxaliplatin**<br>**Gemcitabine/cisplatin**<br>**gemcitabine**	Unable to treat patients with biliary tract cancers with current EML. While these medicines are often widely available, and are listed elsewhere in the EML, they are not included in the EML for biliary tract cancers due to the low magnitudes of clinical benefit in this disease.	No change
**Esophageal**	No EML-listed medicines for this indication	No systemic therapy recommended in core resources	Unable to treat any patients with esophageal cancer with current EML. No systemic therapy is recommended in either NCCN on EML.	Consider listing for 5FU and oxaliplatin for metastatic esophageal cancer
**Gastric**	No EML-listed medicines for this indication	No systemic therapy recommended in core resources	Unable to treat any patients with gastric cancer with current EML. No systemic therapy is recommended in either NCCN on EML.	Consider listing for 5FU and oxaliplatin for metastatic gastric cancer
**HCC**	No EML-listed medicines for this indication	No systemic therapy recommended in core resources	No systemic therapy is recommended in guidelines or EML. Limited high value affordable therapies	No change
**Kidney**	No EML-listed medicines for this indication	**Axitinib/pembrolizumab**<br>**Cabozantinib/nivolumab**<br>**Lenvatinib/pembrolizumab**<br>**Ipilimumab/nivolumab**<br>**Cabozantinib**<br>**Sunitinib**<br>**Nivolumab**	No EML-recommended medicines. NCCN-listed medicines are expensive and generally of low value, warranting exclusion from EML.	No change

**Pancreas Cancer**	No EML-listed medicines for this indication	**5-Fluorouracil/oxaliplatin/ irinotecan/calcium folinate**<br>**Gemcitabine/capecitabine**<br>**Capecitabine**<br>**Gemcitabine**	Guideline listed treatments generally fail to meet EML thresholds of benefit.	No change
**Thyroid Cancer**	No EML-listed medicines for this indication	No systemic therapy recommended in core resources	No EML-listed medicines recommended in guidelines or EML. Limited high-value affordable therapies	No change
**Uterine cancer**	No EML-listed medicines for this indication	**Doxorubicin (sarcoma only)**<br>**Carboplatin/paclitaxel**<br>**Medroxyprogesterone**<br>**Tamoxifen**<br>**Megestrole acetate**	Several medicines included as recommended treatment in guidelines include cheap and readily available hormonal therapies (tamoxifen, megestrol acetate and medroxyprogesterone). These medicines should be considered by the EML for inclusion. Furthermore, first-line chemotherapy with carboplatin and paclitaxel is also cheap, available and offers clinical benefit and should be considered by the EML for inclusion.	Consider common hormonal therapies and chemotherapeutic agents including carboplatin and paclitaxel.
